# The Comparative Efficiency of Local Anæsthetics
1Received for publication August, 1925.


**Published:** 1925

**Authors:** Eric Watson-Williams

**Affiliations:** Clinical Lecturer in Laryngology and Otology in the University of Bristol


### THE COMPARATIVE EFFICIENCY OF LOCAL ANÆSTHETICS. [1]

BY

ERIC WATSON-WILLIAMS, M.C., Ch.M., F.R.C.S.E.,

*Clinical Lecturer in Laryngology and Otology in the University of Bristol.*

### INTRODUCTION.

THE synthetic chemist is frequently urged to produce new local anæsthetics better than any we now possess. When he turns to the clinician to learn what properties are to be sought in an ideal drug of this nature he finds, in addition to questions of cost and those side actions which may influence use in certain situations, some such list as the following (2, 7, 11) :—

1. High anæsthetic efficiency.
2. Low toxicity.
3. Absence of irritation.
4. Stability and ready sterilisability.
5. No tendency to "idiosyncrasy."
6. No tendency to "addiction."

As is perhaps natural from those who are bringing out such a drug as a commercial proposition, the defects of the well-known drugs, and especially cocaine, in these categories are apt to receive undue emphasis.

[1] Received for publication August, 1925.

STABILITY.

Thus, with regard to (4), cocaine hydrochloride is not appreciably less stable than its synthetic rivals. All can be sterilised by boiling, if this is done properly ; all are decomposed by prolonged boiling or by alkalies, and afford a ready pabulum to moulds if carelessly stored. It is an open question whether great stability is a desirable property —it might well lead to increased difficulty on the part of the patient in the disposal of the drug after use.

"IDIOSYNCRASY."

It is often assumed that patients exhibit "idiosyncrasy" to cocaine only. This is not so ; novocaine, alypine and stovaine have all given rise to this trouble at times. That is to say, some degree of disturbance has been caused by a dose small and usually free from this risk. If by "idiosyncrasy" we imply that doses ordinarily safe may in certain individuals cause death, we are discussing a phenomenon that on the face of it is so rare as to be a pathological curiosity. Examination of the evidence demonstrates how very inadequate it is ([8, 9, 13]). But it is to be remarked that the readiness with which cocaine gives signs of overdose—with a dose far short of the fatal dose—so far from being a disadvantage is one of the best possible safeguards. Indeed, this is probably the only reason why fatal accidents have in forty years of universal use been of extraordinary rarity. Elsewhere I have brought evidence on this point, and the close connection between this property and "addiction" ([16]). The social disadvantages ([4, 5]) of the latter phenomenon must be trivial in comparison with the boon of clinical security.

### CRITERIA OF EFFICIENCY.

We have thus remaining the first three postulates only to consider. The position may be re-stated thus :—

(*a*) The ideal local anæsthetic should induce complete loss of function in the sensory nerve endings, fibres or cells with which it comes in contact, and that in a concentration at which it does no appreciable damage to other tissues.

In order to fulfil this demand a drug must have some special "selective" action on sensory nerve tissue ([4]). It is true that the action of some of these drugs is not the same for all types of nervous tissue; they affect, as a rule, sensory fibres more readily than motor ([3]). But this is a matter of degree only; the effect of such drugs on other nerves is not materially less than that on sensory nerves, when compared with their effect on other tissues. We can, therefore, hardly expect that a drug that will readily paralyse sensory fibres will not also paralyse the nervous tissue of the medulla, if present in sufficient concentration ([10, 15]). The second criterion of efficiency may therefore be stated :—

(*b*) The ideal local anæsthetic should be non-poisonous; in the sense that the dose necessary for (*a*) is very far short of the dose that, gaining entrance to the circulation, will paralyse the medulla.

The efficiency of any drug as a local anæsthetic is for practical purposes determined by the way in which it fulfils these two criteria. It is, therefore, interesting to consider what are the means we adopt to discriminate between different drugs as to their efficiency.

### ANÆSTHETIC EFFICIENCY.

A variety of means has been adopted to compare the anæsthetic powers of various drugs. It has long been recognised that animal experiments are of little real value on this point, often misleading and difficult of clinical application (7). But the value of various methods of investigation in humans is by no means identical. The simplest method of test is to apply under the same conditions, for the same arbitrarily-fixed time, solutions of the same arbitrarily-fixed strength, and to assign values in accordance with the production or otherwise of good anæsthesia. Since there is no certainty that the conditions selected are suitable for the action of all the drugs, it is not to be wondered at that very various results are obtained. A similar method is based not on the production or otherwise of anæsthesia, but on its duration (12); the latter depends on a number of factors, among them the total quantity of drug present in a given area at any time. It leads to the conclusion, for example, that one volume of 2 per cent. solution of cocaine does *not* contain approximately twice as many "anæsthetic units" as the same volume of 1 per cent. solution (2), a *reductio ad absurdum*.

### METHOD OF COMPARISON.

The method that I have found most satisfactory in comparing different drugs of this class is as follows. We determine *in what concentration* the same volume of each solution will produce complete anæsthesia *in the same time*. It is then fair to assume that each solution contains the same number of "anæsthetic units" per c.c. Working on these lines, one obtains results as follows:—

TABLE A.

| Drug (Surface application). (*a*) | Concentration, per cent. | Time required to induce anæsthesia, seconds. (*b*) | Concentration of cocaine inducing anæsthesia in same time, per cent. | Comparative anæsthetic value Cocaine = 1. |
|---|---|---|---|---|
| Cocaine HCl [18] . . | 2 | 483 | — | — |
| | 3 | 268 | — | — |
| | 5 | 125 | — | — |
| | 10 | 45 | — | — |
| Novocaine . . . . | 20 | 411 | 2·2 | 0·11 |
| Alypine . . . . | 5 | 325 | 2·6 | 0·52 |
| | 10 | 133 | 4·8 | 0·48 |
| Stovaine . . . . | 10 | 171 | 4·0 | 0·40 |
| Benzamine . . . . | 10 | 240 | 3·2 | 0·32 |
| Methyl-benzamine (*c*) | 5 | 425 | 2·2 | 0·44 |
| Psicaine [14] . . . . | 5 | 212 | 3·5 | 0·70 |
| | 7 | 119 | 5·1 | 0·73 |
| Tutocaine [17] . . | 5 | 208 | 3·5 | 0·70 |
| | 10 | 78 | 6·8 | 0·68 |
| Apothesine . . . . | 10 | 288 | 2·9 | 0·29 |
| Butyn . . . . | 2 | 272 | 3·0 | 1·50 [12] |
| | 5 | 78 | 6·8 | 1·36 |
| Novocaine subcutaneous (*d*) . . | 0·5 | — | 0·1+ | 0·25 |

(*a*) For technique, see [18].

(*b*) Small errors in estimating time produce no appreciable effect on the result.

(*c*) The methyl homologue of benzamine, prepared by the Department of Chemistry, Bristol University.

(*d*) Inserted for comparison.

### COMPARATIVE TOXICITY.

When investigating the effect on the medulla of these drugs it is, of course, not possible to conduct human experiments. We must rely on animal experiments, and assume that the ratio of toxicities found in this way is the same as the ratio of toxicities to man. The value of our experiments is influenced by the species chosen ([6, 11, 16]) and by the fidelity with which we reproduce the conditions which obtain in the clinic. For example, if we employ hypodermic injections, the drug should be in the very low concentration in which it is used clinically—concentration very considerably influences our results ([15]). A series of some 300 experiments has given the results shown in Table B :—

TABLE B.

| Drug. | Experimental lethal toxicity in guinea-pig by subcutaneous injection. kgm. per gm.[15] | | | Comparative toxicity. Cocaine = 1. |
|---|---|---|---|---|
| | ½ per cent. | 1 per cent. | 5 per cent. (*a*) | |
| Cocaine HCl .. .. | 16 | 20 | 25 | — |
| Novocaine .. .. | ? 2·5 | — | 2·5 | 0·16 |
| | — | — | 3 | 0·12 |
| Alypine .. .. | 11·5 | — | — | 0·7 |
| Stovaine .. .. | — | 7 | — | 0·35 |
| Benzamine .. .. | — | 9 | — | 0·45 |
| Methyl-benzamine .. | — | — | 14 | 0·56 |
| Psicaine .. . .. .. | 17 | — | — | 1 + (b) |
| Tutocaine .. .. | 6 | — | — | 0·4 |
| Apothesine .. .. | — | 7 – | — | 0·33 |
| Butyn .. .. .. | 16 | — | — | 1 (b) |

The protocols of these experiments are deposited in the libraries of Bristol and Cambridge Universities and of the Royal Society of Medicine.

(*a*) In this high concentration the relative toxicity appears rather low.

(*b*) The relative toxicity of psicaine and of butyn ([15]) is higher at ¼% concentration.

### EFFICIENCY RATIO.

We are now in a position to see which drugs best fulfil the conditions laid down as desirable. For this purpose we divide the value obtained for anæsthetic efficiency for each drug by that obtained for toxicity.

TABLE C.

| Drug. | Comparative anæsthetic value. Cocaine = 1. (Table A.) | Comparative toxicity. Cocaine = 1. (Table B.) | Cocaine efficiency ratio. |
|---|---|---|---|
| Cocaine HCl .. .. .. | 1 | 1 | 1 |
| Novocaine .. .. .. | 0·11 | 0·12 | 0·9 |
| (injected dilute) .. .. | 0·25 | 0·16 | 1·5 |
| Alypine .. .. .. .. | 0·5 | 0·7 | 0·7 |
| Stovaine .. .. .. .. | 0·4 | 0·35 | 1·1 |
| Benzamine .. .. .. | 0·32 | 0·45 | 0·7 |
| Methyl-benzamine .. .. | 0·44 | 0·56 | 0·8 |
| Psicaine .. .. .. .. | 0·7 | 1 + | 0·7 |
| Tutocaine .. .. .. | 0·7 | 0·4 | 1·7 |
| Apothesine .. .. .. | 0·29 | 0·33 | 0·9 |
| Butyn .. .. .. .. | 1·4 | 1 | 1·4 |

From Table C it appears that the efficiency ratio of all these substances is somewhere about unity. When it is remembered that the two factors concerned have been estimated on very different species, it appears probable that the variations are within the limits of experimental accuracy. If so, we are forced to the conclusion that *the anæsthetic power of all these substances is roughly proportional to their toxicity* ([15]). In other words, the power of each of paralysing sensory nerve fibres is proportional to its power of paralysing the nervous tissue of the medulla. Therefore, as regards their comparative efficiency, there is little to chose between them. Certain of them may have special advantages for particular types of work; some of them may have disadvantages as being irritant. But safety in use is governed by the considerations set forth in the beginning, and not by any marked difference in the power of an effective anæsthetic dose of any one of them to kill. Until some radical departure from the type of compound at present in use is attained it appears vain to expect an anæsthetic which is materially better (considering both efficiency and safety) than cocaine.

### SUMMARY.

1. All "local anæsthetics" are efficient if sufficient time is allowed for them to act, and the concentration employed reaches the necessary minimum.

2. All "local anæsthetics" are safe, in the sense that, used with care and intelligence, the risk of death should be negligible.

3. No "local anæsthetic" is safe in the sense that ignorant or careless use involves no serious risk of disaster.

4. Those drugs which give rise to disturbance in a dosage far short of a fatal dose, though possibly less convenient than others, are likely to be the safest in ordinary practice.

5. There appears no theoretical reason why those who have learnt to use cocaine, either by injection or by surface application, should not continue to do so. None of its rivals appear to have *any material advantage* over it from *any point of view.* We cannot at present hope that any compound with a similar mode of action will ever possess such advantage.

### ACKNOWLEDGMENT.

The expenses of this research have been met by a grant from the Colston Research Fund of Bristol University, for which my thanks are due. I am also indebted for permission to work in the Pathological Department of Bristol University to Professor Walker Hall; and for supplies of synthetic drugs to Professor F. Francis; the Abbott Laboratories, Chicago; Messrs. Merck, Darmstadt; Messrs. Bayer, Ludwigshafen; and Laboratoire L. Schaerer, Paris.

REFERENCES.

[1] Bonar and Sollmann, *J. Pharmacol.*, 1921, xviii. 467.
[2] Copeland, *Brit. M. J.*, July 12th, 1924, p. 41.
[3] Dixon, *J. Physiology*, Dec. 30th, 1904, lvi. 87.
[4] *Ibid.*, *Proc. Roy. Soc. Med.*, June, 1924.
[5] *Ibid.*, *Brit. M. J.*, 1924, ii. 765.
[6] *Ibid.*, *Manual of Pharmacol.*, 1921.
[7] Le Brocq, *Brit. M. J.*, March 27th, 1909, p. 783.
[8] Mayer, *J. Amer. M. Ass.*, 1920, p. 315.
[9] *Ibid.*, 1923, p. 947.
[10] Meeker and Frazer, *J. Pharmacol.*, 1924, xxii. 387.
[11] Neilsen and Higgins, *J. Lab. and Clin. Med.*, 1923.
[12] Schmitz and Loevenhart, *J. Pharmacol.*, 1924, xxiv. 159, 167.
[13] E. W.-W., *Brit. M. J.* Dec. 1st, 1923, p. 1018.
[14] *Ibid.*, Jan. 3rd, 1925.
[15] *Ibid.*, *Proc. Roy. Soc. Med.*, 1924, xvii. 77.
[16] *Ibid.*, *Med. Press*, March 18th, 1925, p. 211.
[17] *Ibid.*, *Lancet*, May 2nd, 1925, p. 913.
[18] *Ibid.*, *J. Laryngol.*, June, 1925, p. 374.

